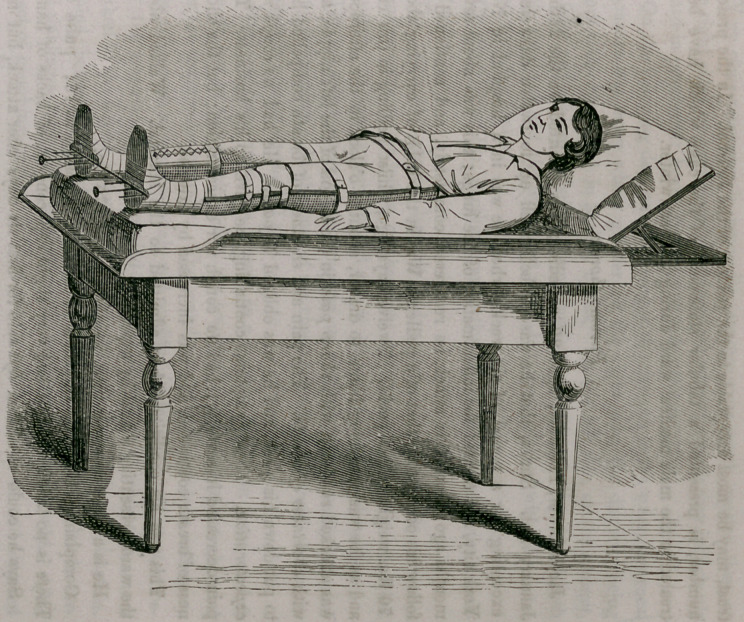# Wire Cloth in Orthopædic and General Surgery

**Published:** 1857-04

**Authors:** Louis Bauer

**Affiliations:** Corresponding Fellow of the London Med. Society; Resident Fellow of the New York Pathological and German Med. Chirurgical Society


ART. II.—*Wire Cloth in Orthopædic and General Surgery.* By Louis Bauer, M. D., M. R. C. S. E.; Corresponding Fellow of the London Med. Society; Resident Fellow of the New York Pathological and German Med. Chirurgical Society.

During the last four years I have successfully used wire for orthopædic purposes. In the first place I had frames made of thin steel or iron, and had the space filled up with copper wire braided. The process of making this apparatus, however, was rather slow and expensive, and very soon, therefore, the brass and copper wire cloth suggested itself, as, in every respect, preferable. Since two years, however, I have exclusively applied iron wire cloth, being tinned by a galvanic process, and subsequently varnished, in order to keep it from rusting.

The pliability of this material renders it exceedingly useful for surgical purposes, and during the above mentioned period I have had made splints of it and appropriated them in the dressing of fractures. At various occasions they were submitted to the inspection of the German Med. Chirurg. and Pathological Society of New York, and this circumstance is sufficient to secure to me the priority of the invention, at least over my esteemed friend, Dr. J. C. Nott, of Mobile, who suggested the usefulness of wire cloth at a much later period. The application of wire cloth in the Crimean war falling in the same time of my invention, was unknown to me.

Moreover, in the Crimea as well as by Dr. Nott, the wire cloth is used alone, and not with the addition of frames, which latter I consider of practical importance: for they give a greater firmness and solidity to the fracture-splint without interfering in any way with its good qualities.

These splints are made upon gypsum or wood-casts, of different sizes, in the same manner as Dr. Welsh's splints seem to be manufactured, so that they exactly correspond with the shape of the member. Small differences between the forms may be soon remedied by a single bend.

Compared with other material hitherto in use in dressing fractures, wire splints are decidedly superior. They are exceedingly light and permeable, keeping the limb cool and permitting the application of fomentations, warm or cold, without losing in their firmness; and they are, therefore, exceedingly useful in combination with the so-called "permanent bath," of Professor Langenbeck, of Berlin. The way in which I apply them in fractures, is exceedingly simple and expeditious. After the bones have been properly set, I fill both splints with loose cotton wool, and fasten them by a roller to the extremity. In a number of instances in my own hands and those of medical friends, this mode of dressing fractures has answered every expectation. I have never observed undue inflammation or swelling, and if they should take place the cotton wool would compress and sufficiently yield the requisite room, without demanding the removal of the dressing. Whether the peculiar property of cotton wool, or the great coolness or permeability of the splints, prevented in my cases the excess of inflammation and swelling I can not as yet say, but the facts seem to be pretty evident.

The same material I have appropriated, also, for an apparatus useful and decidedly superior to all those hitherto in use for similar purposes, in the following cases:

1. Incipient hip disease, in order to secure absolute rest of the affected joint, and a proper position of the corresponding extremity, with a view of preventing deformity, the almost unavoidable consequence of hip disease.

2. Dislocation of the hip-joints in order to retain the reduced head of the femur in the acetabulum, and to protect it against accidental movement.

3. In fractures of the neck and shaft of the femur. In the latter case it is particularly useful and applicable, but more especially for young patients, because it offers a safe and soft bed; it permits the passage of evacuation, without any trouble or change in the position of the patient; it divides the contra-extension between the healthy extremity and the tubera ischii, and can be borne for months without danger of bed-sores and excoriations; it permits any amount of extension upon the fractured extremity, and, in fine, it allows even the patient to be carried about or to ride in a carriage, without danger of displacing the fragments of the fractured bone.

Dzondi and Hagedorn's fracture apparatus has served as the prototype of my construction, but the most superficial comparison cannot fail in discovering the superior advantages of my apparatus over the former.

My apparatus consists of a thick wire frame, similating in its form the posterior half of a pair of pants. Corresponding with the anus a sufficiently large piece is removed for alvine evacuations. Both legs are kept apart by an iron bar, through which, on either side, three holes are made. The middle one, the widest, is designed to receive a stout screw, and the two lesser openings a small piece of stoutish wire. The three being riveted to an iron foot board, serve to move the said foot board up and down, in all, perhaps, a space of three inches.

In order to place the patient into my apparatus for any of the aforementioned objects, it is to be filled with loose cotton wool, and the calf of the affected or fractured limb, is to be surrounded by a lace stocking.

So prepared, the patient is put into the apparatus; the foot of the sound member is to be firmly set upon the foot board, the limb stretched and kept in this position with bandage and knee cap, surrounding both the whole limb and the instrument, thus preventing the said limb from flexing. The fracture is then adjusted and the extension made by buttoning the lace stocking to the foot board, the foot being bandaged on the latter. By means of the screw the degree of extension must be regulated. The thigh is then to be covered with a small wire splint in the front, and both the splint and limb fastened by a roller upon the apparatus; and, finally, the pelvis is kept down by a belt. This is the whole, and it will be easily perceived that the limb, thus secured, cannot move in the least, or the fractured bone be displaced. For the purpose of alvine evacuations, the lower portion of the apparatus is so much raised as to permit the placing of a bed-pan under the posteriors, which can be done without any apprehension concerning the security of the limb.

The following diagrams will illustrate both my splints and apparatus.

## Figures and Tables

**Figure f1:**
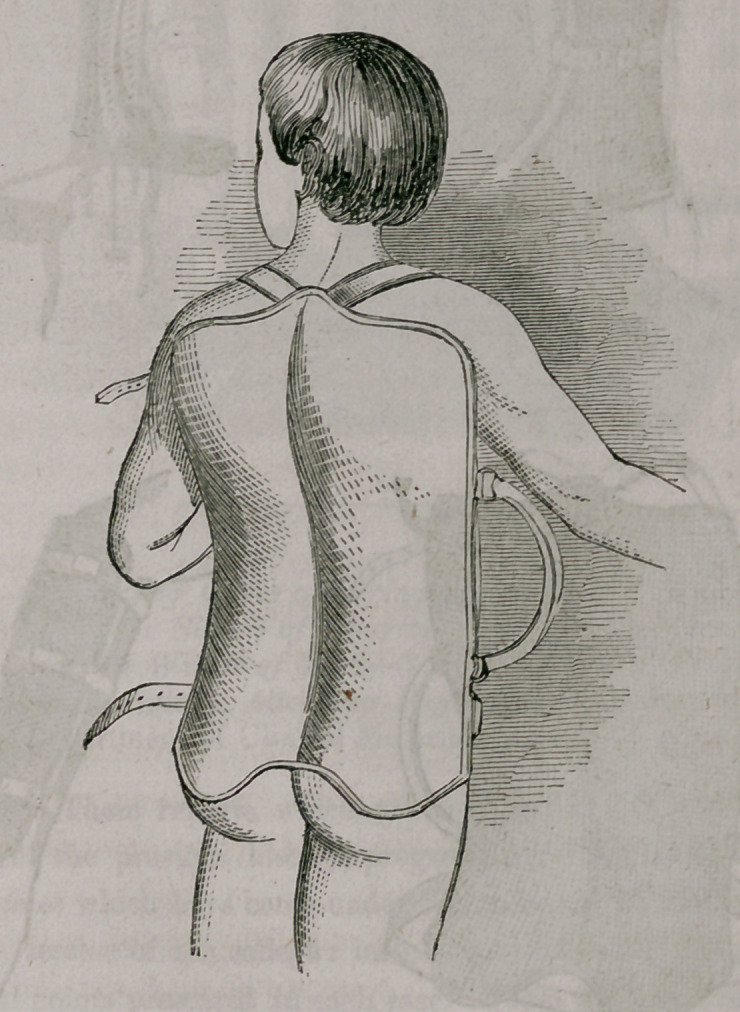


**Figure f2:**
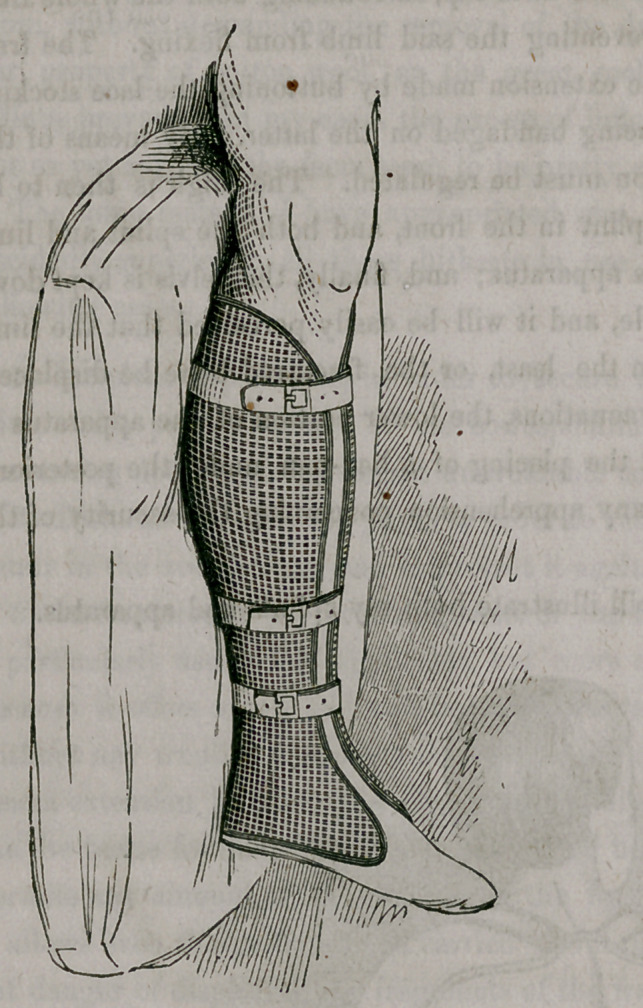


**Figure f3:**
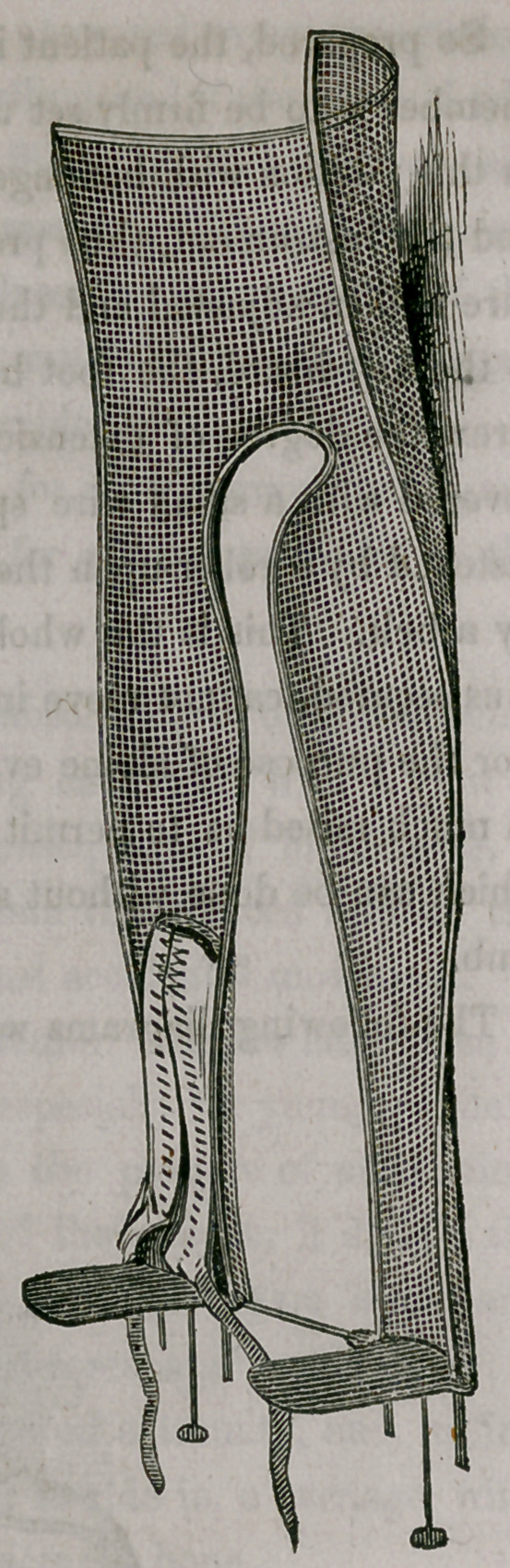


**Figure f4:**
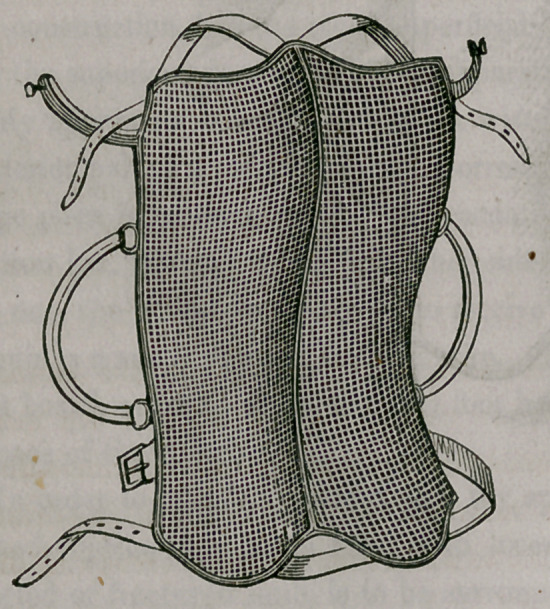


**Figure f5:**
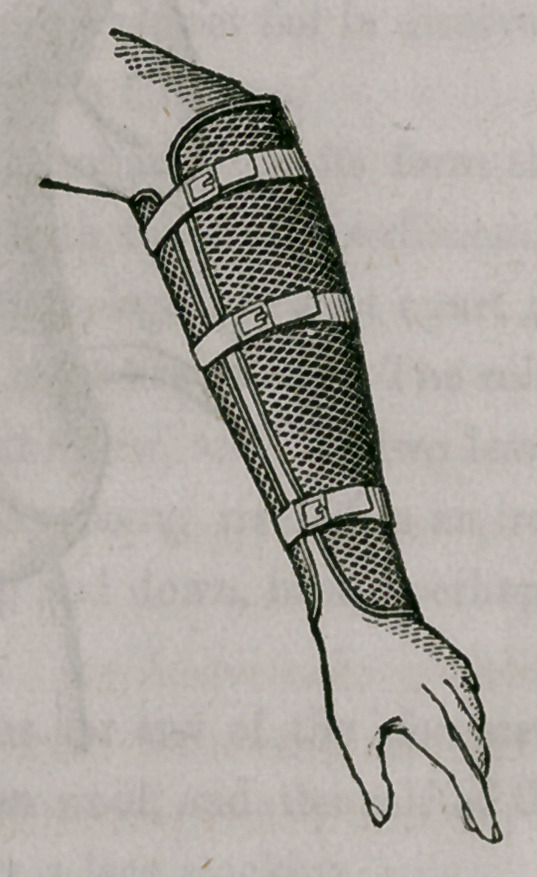


**Figure f6:**